# Prevalence of elevated serum fatty acid synthase in chronic limb-threatening ischemia

**DOI:** 10.1038/s41598-021-98479-7

**Published:** 2021-09-29

**Authors:** Shirli Tay, Gayan S. De Silva, Connor M. Engel, Nikolai Harroun, Amanda S. Penrose, Kshitij A. Desai, Yan Yan, Clay F. Semenkovich, Mohamed A. Zayed

**Affiliations:** 1 Section of Vascular Surgery, Department of Surgery, Washington University School of Medicine, St. Louis, MO USA; 2 Division of Public Health Sciences, Department of Surgery, Washington University School of Medicine, St. Louis, MO USA; 3 Division of Endocrinology, Metabolism and Lipid Research, Department of Internal Medicine, Washington University School of Medicine, St. Louis, MO USA; 4 Division of Molecular Cell Biology, Washington University School of Medicine, St. Louis, MO USA; 5 Department of Biomedical Engineering, Washington University, McKelvey School of Engineering, St. Louis, MO USA; 6 Veterans Affairs St. Louis Health Care System, St. Louis, MO USA

**Keywords:** Enzyme mechanisms, Enzymes, Histocytochemistry, Lipids, Proteins, Biomarkers, Diseases, Pathogenesis

## Abstract

There are currently no serum-based evaluations that can corroborate the severity of peripheral artery disease (PAD). In this cross-sectional study, we assessed the prevalence of elevated serum fatty acid synthase (cFAS) in patients with chronic limb-threatening ischemia (CLTI) and evaluated the accuracy of its use in detecting this condition. Preoperative fasting serum samples from 87 patients undergoing vascular intervention were collected between October 2014 and September 2016. Median age was 62 years, with 56 (64%) men, and 32 (37%) with CLTI. We found that elevated cFAS content (OR 1.17; 95% CI 1.04–1.31), type 2 diabetes (T2D; OR 5.22; 95% CI 1.77–15.4), and smoking (OR 3.53; 95% CI 1.19–10.5) were independently associated with CLTI and could detect the presence of CLTI with 83% accuracy (95% CI 0.74–0.92). Furthermore, serum FAS content was positively correlated with FAS content in femoral artery plaque in patients with severe PAD (${R}^{2}$ = 0.22; *P* =  0.023). Finally, significantly higher co-localization of FAS and ApoB were observed within lower extremity arterial media (*P* < .001). Our findings indicate that serum FAS content is a marker for disease severity in patients with PAD, independent of concomitant T2D and smoking, and may play a key role in FAS and ApoB peripheral plaque progression.

## Introduction

Peripheral artery disease (PAD) impacts 12 million people in the US^1^. Among them at least 1 in 10 will progress to develop chronic limb-threatening ischemia (CLTI)^2^, a condition characterized by severe lower extremity arterial insufficiency, rest pain, non-healing wounds/ulcers, and gangrene^3,4^. Treatment of incident cases of CLTI is associated with high costs that exceed $100–300 million per year^2,5–7^. Despite the high level of management required for patients with CLTI, it is reported that up to 70% will still develop disease-related complications, and ultimately receive an extremity amputation^8,9^. In order to improve prognosis, the Global Vascular Guidelines recently highlighted the need for early diagnosis and aggressive medical management of patients with CLTI—particularly among those with cardiovascular risk factors such as type 2 diabetes (T2D)^3^. Therefore, an accurate biochemical serum diagnostic strategy that can identify individuals with PAD who are at higher risk of progression to CLTI would be immensely beneficial for prompt diagnosis and treatment in order to reduce disease-related morbidity^3^.

Fatty acid synthase (FAS) is an essential enzyme that regulates de novo lipogenesis in various organ systems including cardiovascular tissue^10–12^. We previously discovered that serum circulating FAS (cFAS) was elevated in patients with atherosclerotic carotid artery stenosis, and FAS content in carotid plaque was higher in maximally diseased segments^13^. Interestingly, it was also observed that cFAS predominately fractionated with serum LDL cholesterol and co-immunoprecipitated with apolipoprotein B (ApoB; the dominant protein in LDL particles)^14,15^. These findings suggested that LDL may be a cFAS carrier that distributes it to peripheral arterial tissue that is prone to atheroprogression^13^, and that cFAS may be an important marker for disease severity.

Here we sought to determine whether serum cFAS could accurately distinguish between patients with or without CLTI. We hypothesized that higher serum cFAS is an indicator of CLTI, and that FAS localized to peripheral arterial tissue is associated with higher atherosclerotic plaque burden.

## Results

### Patient characteristics

**Figure 1:**
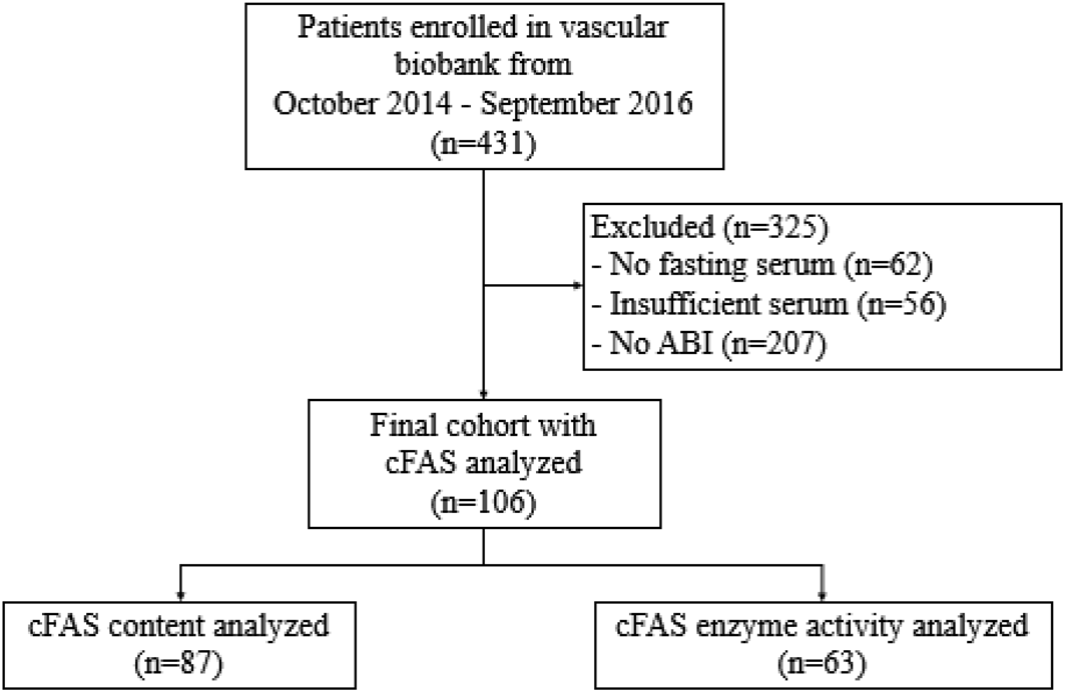
Flow diagram. Between October 2014 and September 2016, 431 patients were enrolled in the Washington University Vascular Surgery Biobank Repository. Patients who did not provide fasting serum (n = 62), had missing ABI (n = 207), or insufficient serum samples (n = 56) were excluded from this study. A total of 87 patients were included in this study and underwent analysis for cFAS content, while 63 patients underwent analysis for cFAS enzyme activity.

From October 2014 to September 2016, 431 patients were enrolled in the Washington University School of Medicine Vascular Surgery Biobank. Patients excluded from this study (n = 325) did not provide adequate volume of fasting serum or did not have a recorded ankle-brachial index (ABI) within 3 months of their planned elective operative intervention (Fig. 1). Among the 87 patients who were included in this study the median age was 62 years (IQR 49–68 years) with 56 (64%) male, 71 (82%) Caucasian, 32 (37%) had CLTI, and 32 (37%) had T2D (Table 1).

**Table 1:** Baseline characteristics of patients who underwent analysis for serum FAS content.

Characteristics	All patients n = 87	CLTI n = 32	No CLTI n = 55	P value
cFAS content	4.11 (2.29–7.48)	7.03 (3.33–10.33)	3.02 (1.86–5.45)	< 0.001*
Age, median—years	62 (49–68)	63 (59–69)	59 (30–67)	0.007*
**Sex—no. (%)**				0.78^†^
Male	56 (64.4)	20 (62.5)	36 (65.5)	
Female	31 (35.6)	12 (37.5)	19 (34.6)	
BMI, median—kg/m^2^	29.4 (23.5–33.1)	26.8 (22.7–34.3)	29.7 (25.3–32.3)	0.66*
**Race—no. (%)**				0.074^†^
Caucasian	71 (81.6)	23 (71.9)	48 (87.3)	
African American	16 (18.4)	9 (28.1)	7 (12.7)	
**T2D—no. (%)**	32 (36.8)	20 (62.5)	12 (21.8)	< 0.001^†^
DCSI score ≥ 5	22 (68.8)	14 (70)	8 (66.7)	1^‡^
Insulin use	15 (46.9)	11 (55)	4 (33)	0.29^‡^
**CKD—no. (%)**	33 (37.9)	15 (46.9)	18 (32.7)	0.19^†^
CKD 1–3	28 (84.8)	11 (73.3)	17 (94.4)	
CKD 4–5	5 (15.2)	4 (26.7)	1 (5.6)	
Hyperlipidemia—no. (%)	40 (46)	18 (56.3)	22 (40)	0.14^†^
HTN—no. (%)	55 (63.2)	26 (81.3)	29 (52.7)	0.008^†^
CHF—no. (%)	13 (14.9)	7 (21.9)	6 (10.9)	0.22^‡^
CAD—no. (%)	29 (33.3)	9 (28.1)	20 (36.4)	0.43^†^
CAR—no. (%)	11 (12.6)	3 (9.4)	8 (14.6)	0.74^‡^
COPD—no. (%)	14 (16.1)	8 (25)	6 (10.9)	0.085^†^
**Smoking status—no. (%)**				0.020^†^
Never/former	57 (65.5)	16 (50)	41 (74.6)	
Current	30 (34.5)	16 (50)	14 (25.4)	
**PAD status—no. (%)**				N/A
No PAD	28 (32.2)	–	28 (50.9)	
Rutherford 0	4 (4.6)	–	4 (7.3)	
Rutherford 1	0	–	0	
Rutherford 2	2 (2.3)	–	2 (3.6)	
Rutherford 3	21 (24.1)	–	21 (38.2)	
Rutherford 4	17 (19.5)	17 (53.1)	–	
Rutherford 5	9 (10.3)	9 (28.1)	–	
Rutherford 6	6 (6.9)	6 (18.8)	–	
**Procedure—no. (%)**				N/A
TOS/venous stab	20 (23)	0	20 (36.4)	
AAA/TAA repair	17 (19.5)	2 (6.3)	15 (27.3)	
CEA/CAS/dialysis access	6 (6.9)	1 (3.1)	5 (9)	
LE interventions	44 (50.6)	29 (90.6)	15 (27.3)	

AAA: abdominal aortic aneurysm; BMI: body mass index; CAR: carotid artery stenosis; CAS: carotid artery stenting; CEA: carotid endarterectomy; cFAS: serum circulating fatty acid synthase; CHF: congestive heart failure; CKD: chronic kidney disease; CLTI: chronic limb-threatening ischemia; COPD: chronic obstructive pulmonary disease; Current smoker: last smoked within 12 months of vascular procedure; DCSI: diabetes complications severity index; Former smoker: last smoked > 12 months before procedure; HTN: hypertension; LE interventions: lower extremity interventions included endovascular or open procedures and amputation; PAD: peripheral artery disease; T2D: type 2 diabetes mellitus; TAA: thoracic aortic aneurysm; TOS: thoracic outlet syndrome; N/A: not applicable; Continuous variables are presented as medians with interquartile ranges. Categorical variables are presented as frequencies and percentages.

*Mann–Whitney U-test. ^†^Chi-square test. ^‡^Fisher’s Exact test.

At baseline, patients with CLTI had higher levels of serum cFAS content (7.03 *vs*. 3.02; *P* < 0.001), were older (63 *vs.* 59 years; *P* = 0.007), more likely to be current smokers (50% *vs.* 25%; *P* = 0.02), have concomitant T2D (63% *vs.* 22%; *P* < 0.001), and hypertension (81% *vs.* 53%; *P* = 0.008) compared to patients without CLTI. Serum cFAS enzyme activity was analyzed in a subset of 63 patients but no significant difference was found between CLTI and non-CLTI patients (0.65 *vs.* 0.54 pmol; *P* = 0.75).

### Independent factors and accuracy of detecting CLTI

**Figure 2:**
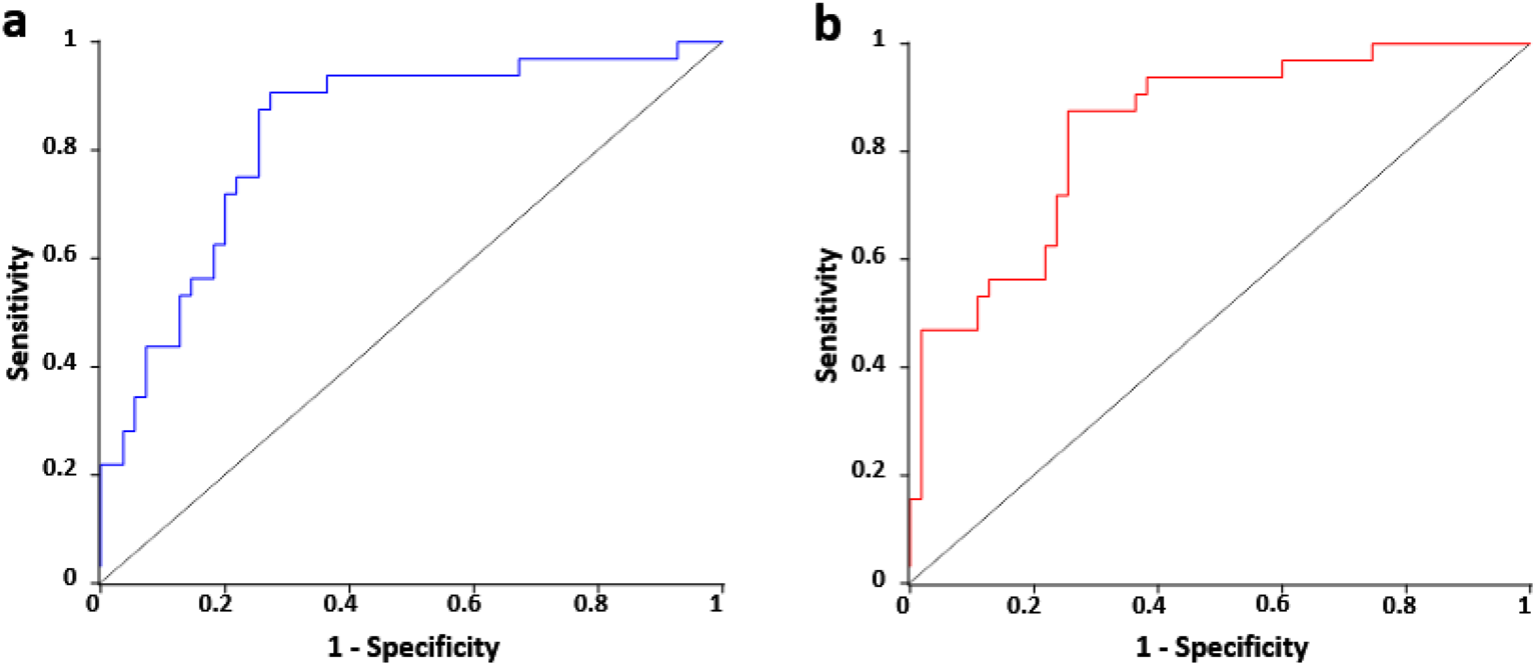
Distinguishing CLTI using serum FAS content and other variables. (**a**) Area under the curve containing serum cFAS content, T2D, and smoking status demonstrates 83% accuracy in distinguishing CLTI patients. (**b**) When adjusted for age and sex, accuracy improved marginally to 84%. Diagonal line represents random probability (no distinguishing ability).

On multivariable analysis, CLTI was found to be significantly associated with cFAS content (OR 1.17; 95% CI 1.04–1.31), T2D (OR 5.22; 95% CI 1.77–15.4), and smoking (OR 3.53; 95% CI 1.19–10.5; Supplementary Table 1, Fig. 1). When adjusted for age and sex, cFAS content (OR 1.16; 95% CI 1.02–1.31), T2D (OR 3.70; 95% CI 1.21–11.3), and smoking (OR 3.32; 95% CI 1.11–9.96) remained significant. Area under the receiver-operating characteristic curve (AUC) demonstrated that cFAS content, T2D, and smoking status were able to distinguish CLTI patients with 83% accuracy (95% CI 0.74–0.92; Fig. 2a). When adjusted for age and sex, the model improved marginally to 84% (95% CI 0.76–0.93; Fig. 2b). There were no significant interactions and Hosmer–Lemeshow * χ*^2^ test demonstrated proper fit (*P* = 0.17).

Multivariable logistic regression comparing patients without PAD (n = 20) and patients with CLTI (n = 32) showed no significant association with cFAS content, T2D, or smoking. When comparing patients with Rutherford classes 0–3 (n = 27) and Rutherford classes 4–6 (n = 32), only cFAS content was significant (OR 1.14; 95% CI 1.01–1.29).

Sensitivity analysis using data from patients with Rutherford classes 0–6 (n = 59) showed that cFAS content (OR 1.11; 95% CI 1.02–1.21) and T2D (OR 3.5; 95% CI 1.30–9.41) significantly increased the odds of having higher Rutherford classes (Supplementary Table 2). Notably, while this was true for most Rutherford class comparisons, there was no significant difference between Rutherford classes 0–3 versus 4–6. Smoking was not associated with the odds of higher Rutherford classes (*P* = 0.5).

### Serum cFAS was not associated with T2D

Due to the high concomitant rate of T2D with CLTI, we performed a secondary analysis to determine whether serum cFAS content was associated with T2D. Among 32 patients with T2D, 22 (69%) patients had diabetes complications severity index (DCSI) score ≥ 5 and 15 (47%) were dependent on insulin (Supplementary Table  3). Multivariable analysis demonstrated that T2D was significantly associated with CLTI (OR 14; 95% CI 2.8–68) and CKD (OR 40; 95% CI 8.3–196). To better determine the effects of cFAS content on T2D, we removed CLTI as a variable and observed that T2D alone was associated with age (OR 1.06; 95% CI 1.01–1.11), sex (OR 0.18 for male; 95% CI 0.04–0.83), and CKD (OR 24; 95% CI 5.9–98.2). Notably, serum cFAS content was not associated with T2D (OR 1.06; 95% CI 0.95–1.19; Supplementary Table 4). Finally, we found no significant difference in cFAS enzyme activity between patients with and without T2D (0.56 *vs.* 0.56 pmol; *P* = 0.7).

### Correlation between serum cFAS and femoral plaque FAS

**Figure 3:**
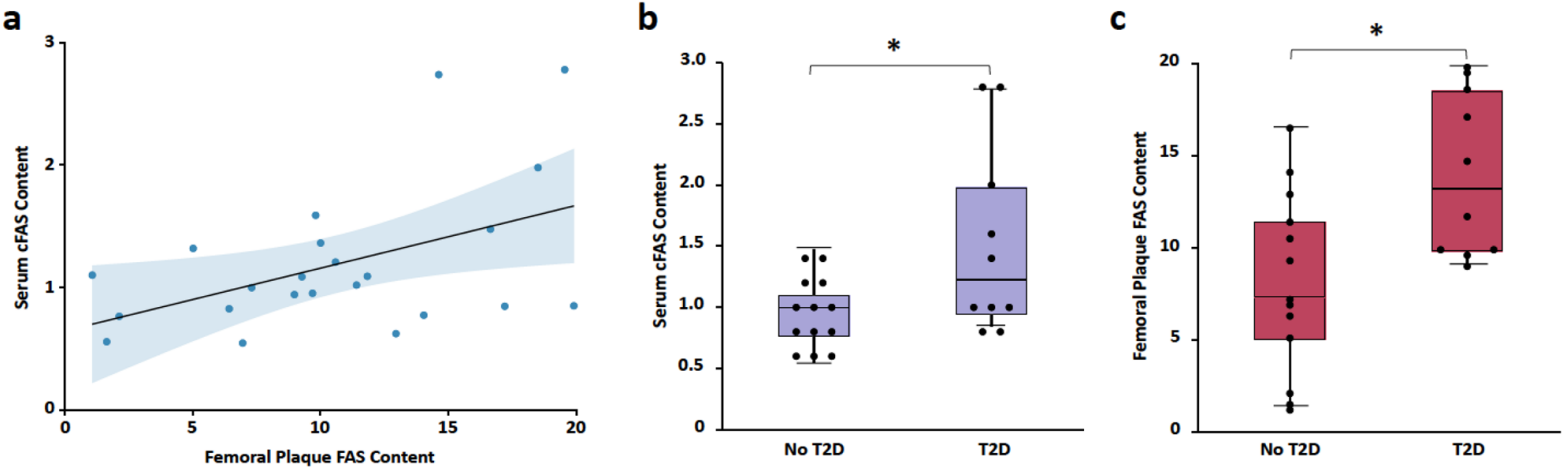
Correlation between FAS content in serum and femoral atherosclerotic plaques of patients with severe PAD*.* (**a**) Regression analysis of FAS content in the serum and plaque of patients with severe PAD demonstrates a significant positive correlation (${R}^{2}$ = 0.22; *P* = 0.023). FAS content is expressed as ratios relative to total serum or plaque protein content. (**b**) Comparison of patients with and without T2D demonstrates that cFAS content was significantly higher in patients with T2D compared to no T2D (1.51 ± 0.75 *vs.* 0.95 ± 0.29; *P* = 0.047). (**c**) Similarly, femoral plaque FAS content was significantly higher in patients with T2D (14.0 ± 4.46 *vs.* 8.11 ± 4.93; *P* = 0.013). Boxplot indicates interquartile range (IQR), median (horizontal line within box), and 1.5 IQR of upper quartile (top whisker) and lower quartile (bottom whisker). cFAS and plaque FAS content were expressed as ratios relative to total serum or plaque protein content, respectively. *P < 0.05.

In a subset of patients with severe PAD (Rutherford 3–5), we evaluated whether serum cFAS content correlated with FAS content in femoral endarterectomy plaque tissue. Among these 23 patients, 16 (70%) were male, 14 (61%) had CLTI, and 10 (43%) had T2D (Supplementary Table 5). Linear regression demonstrated positive correlation between FAS content in the serum and in plaque tissue (${R}^{2}$ = 0.22; *P* = 0.023; Fig. 3a). When comparing patients with and without CLTI (Rutherford 4–5 *vs.* Rutherford 3), there was no significant difference in serum cFAS content (0.9 *vs.* 1.09; *P* = 0.26) or plaque tissue FAS (9.91 *vs.* 10.58; *P* = 0.75; Supplementary Fig. 1). In contrast, when comparing patients with and without T2D, both serum cFAS content (1.51 ± 0.75 *vs.* 0.95 ± 0.29; *P* = 0.047) and plaque FAS (14.0 ± 4.46 *vs.* 8.11 ± 4.93; *P* = 0.013) were significantly higher in patients with T2D (Fig. 3b,c). Subset analysis of patients with T2D demonstrated no difference in serum cFAS content (1.60 ± 0.77 *vs*. 1.43 ± 0.81, *P* = 0.6) and plaque FAS content (12.4 ± 4.1 *vs*. 15.7 ± 4.6, *P* = 0.17) between patients with DCSI score < 5 and ≥ 5 (Supplementary Fig. 2).

### Comparison of free fatty acid content in femoral plaque

Saturated (SFA) and unsaturated free fatty acid (UFA) content were evaluated in femoral endarterectomy plaque specimens from patients with severe PAD (n = 22). Among these patients, 7 patients were Rutherford 3, 13 patients were Rutherford 4, and 2 patients were Rutherford 5. Overall, 10 patients had T2D. We observed no correlation between FFA and FAS content in femoral plaque of patients with severe PAD with or without T2D (Supplementary Table 6). Similarly, there was no correlation between FFA and serum cFAS content in this cohort (Supplementary Table 7).

### Co-localization of FAS and ApoB in lower extremity arterial wall

**Figure 4:**
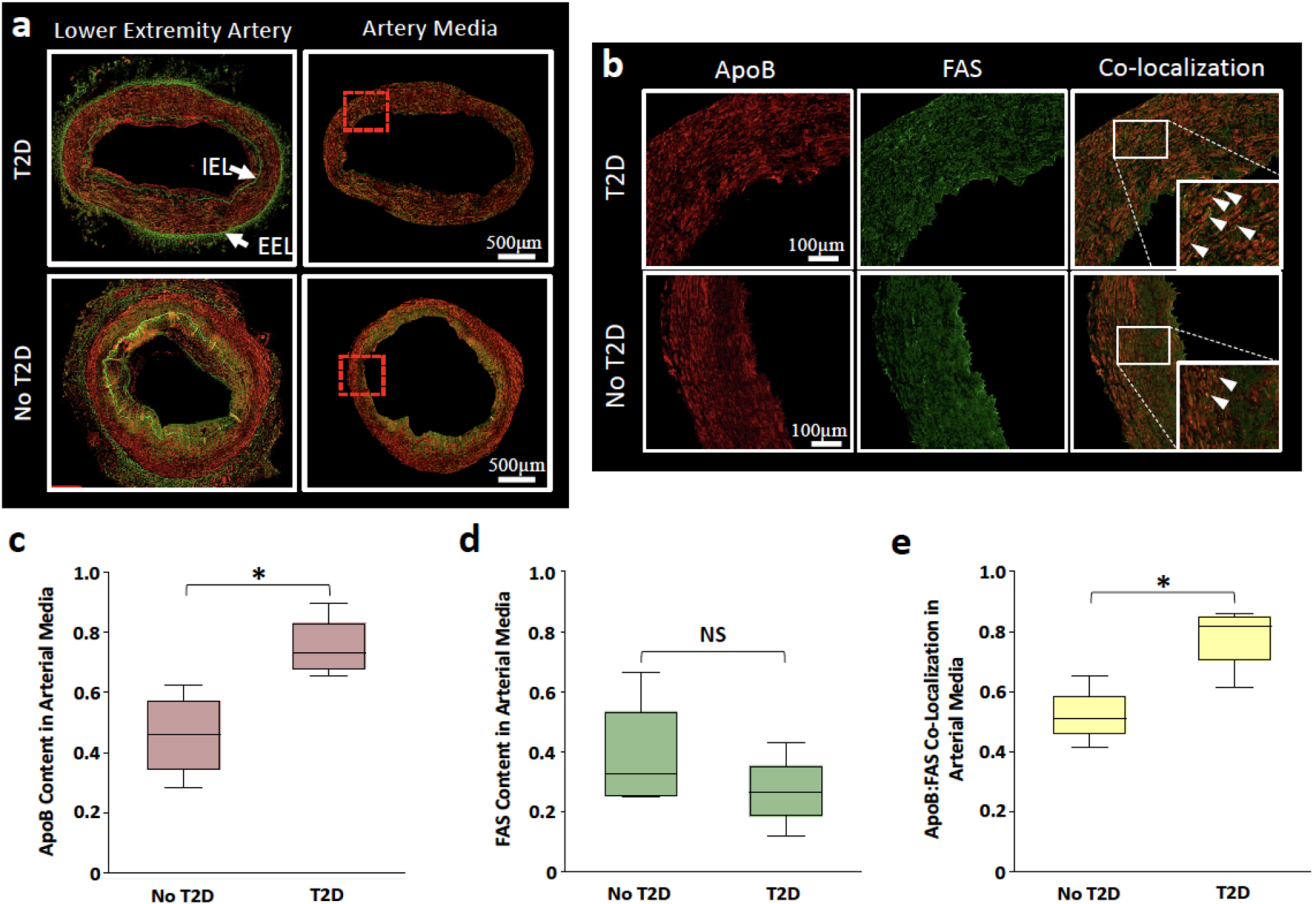
Immunofluorescent staining of FAS and ApoB in lower extremity arterial segments of CLTI patients. Representative lower extremity arterial segments from CLTI patients reflecting the group mean trend were selected. FAS (green), ApoB (red), co-localization of FAS with ApoB (yellow). (**a**) Arterial cross-sections demonstrate arterial intima (lumen boundary to end of IEL) and media (between IEL and EEL). (**b**) ×20 magnification shows co-localization signals (white arrows). (**c**) Mann–Whitney U-test demonstrates that patients with T2D have significantly higher ApoB staining in the arterial media (*P* = 0.021). (**d**) No difference was observed in FAS staining in arterial media of patients with or without T2D. (**e**) ApoB and FAS staining co-localization was higher in patients with T2D (*P* = 0.043). Signals are reported as proportion of total signal type. Boxplot indicates interquartile range (IQR), median (horizontal line within box), and 1.5 IQR of upper quartile (top whisker) and lower quartile (bottom whisker). NS: Not significant. *P < 0.05. IEL: Internal elastic lamina. EEL: External elastic lamina.

To evaluate whether tissue FAS and ApoB co-localize in peripheral arterial tissue, we analyzed arterial wall segments from ischemic amputated lower extremities of patients with CLTI (4 with T2D and 4 without T2D). Randomly selected representative arterial segments reflecting the group mean trends were evaluated (Fig. 4a,b). When compared to CLTI patients without T2D, univariable analysis demonstrated that patients with T2D had significantly higher ApoB content (*P* = 0.021; Fig. 4c) and co-localization of FAS and ApoB (*P* = 0.043; Fig. 4d) in the arterial wall medial layer. However, there was no difference in FAS localization between CLTI patients with and without T2D (*P* = 0.56; Fig. 4e).

**Figure 5:**
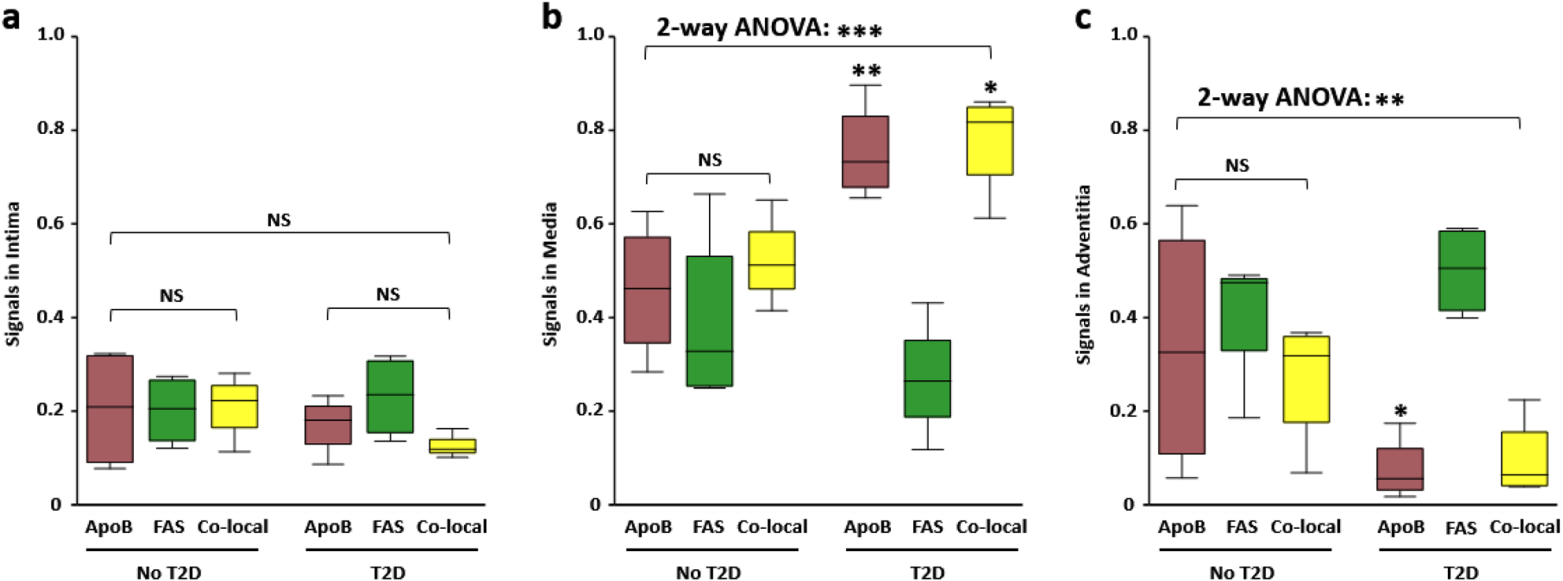
Comparison of FAS and ApoB content and co-localization in lower extremity arterial segments of patients with CLTI. (**a**) One- and two-way ANOVA showed no difference in FAS and ApoB staining intensity was observed in the arterial intima of patients with (*P* = 0.16) and without T2D (*P* = 0.99). (**b**) Two-way ANOVA showed significant interactions between patients with and without T2D (*P* < 0.001). Patients with T2D had significantly higher ApoB (*P* = 0.006) and ApoB:FAS co-localization (*P* = .016) than patients without T2D. No difference was observed in patients with no T2D (*P* = 0*.*51). (**c**) Two-way ANOVA showed significant interactions between patients with and without T2D (*P* = 0.005) that was driven by higher ApoB in patients with no T2D (*P* = 0.026). There was no difference in FAS (*P* = 0.40) or ApoB:FAS co-localization (*P* = 0.13) between patients with and without T2D. Signals are reported as proportion of total signal type. Boxplot indicates interquartile range (IQR), median (horizontal line within box), and 1.5 IQR of upper quartile (top whisker) and lower quartile (bottom whisker). NS: Not significant. *P < 0.05 **P < 0.01, ***P < 0.001.

Two-way ANOVA demonstrated no difference in signals in the intima layer (Fig. 5a). In the media layer, there were significantly higher ApoB and FAS:ApoB co-localization signal (*P* < 0.001) in patients with CLTI and T2D (Fig. 5b). In the adventitia layer, there were significantly higher ApoB signals in patients without T2D (*P* = 0.026), but no difference in FAS (*P* = 0.40) or co-localization signal (*P* = 0.13; Fig. 5c).

## Discussion

We found that serum cFAS content is a marker of CLTI, independent of a concomitant diagnosis of T2D or smoking status. After adjusting for age and sex, each unit increase in serum cFAS content increased the odds of CLTI by 16%, while the presence of T2D and smoking increased the odds of CLTI by 270% and 232%, respectively. In fact, using cFAS content, T2D, and smoking status as markers of disease provided 83% accuracy in distinguishing which patients had CLTI. We also found that for every unit increase in cFAS content, the odds of having a more severe Rutherford classification correspondingly increased by 11%. Notably, when we compared Rutherford classes, we found no difference in cFAS content and T2D between Rutherford classes 0–3 and 4–6, while any other class comparisons yielded significant differences. This shows that serum cFAS content not only correlates positively to increasing severity of PAD, it may also be an important differentiator of impending tissue loss.

In patients with known PAD, CLTI may present when there is clinical progression in disease severity. However, in a large proportion of patients, primary CLTI with no prior diagnosis of PAD can also develop, and T2D appears to be the strongest risk factor for its occurrence (OR 7.45)^2,3^. It is common belief that in patients with primary CLTI, early diagnosis and implementation of aggressive medical management can improve the overall prognosis of the disease. However, patients with CLTI are heterogeneous, and historically this dilemma has contributed to the lack of high-quality epidemiological data on prevalence, incidence, and predictors of CLTI^3^. Further confounding is the observation that patients who undergo amputation as a complication related to CLTI are at increased risk of premature death and are therefore more likely to be missing from population-based studies. Additionally, hemodynamic data required for reliable diagnosis of CLTI can be difficult to obtain and is often lacking in large study populations. A serum-based test for early diagnosis of CLTI and its associated complications is currently missing in the armamentarium of clinicians who presently only rely on low specificity ABI or ultrasound screening techniques.

Our group previously discovered serum cFAS is elevated in individuals with severe atherosclerosis^13^. In a cohort of 39 patients, we observed that cFAS was significantly elevated in individuals with severe carotid artery stenosis and T2D. We found that cFAS correlated not only with serum glucose levels but also with saturated fatty acids in carotid plaque segments. Since patients with carotid artery stenosis have elevated risks of also developing advanced PAD, the current study of serum cFAS in patients with CLTI was a natural extension of our investigation. Here, we similarly observed that serum cFAS content was indeed higher in patients with CLTI. These findings across both studies suggest that elevated serum cFAS in patients with atherosclerosis in the peripheral arterial system (*i.e.,* carotid or lower extremities) is indicative of disease severity.

In tissue, FAS exists as a homodimer of 273 kDa subunits, and is essential for de novo synthesis of saturated fatty acids from simple precursors such as acetyl-CoA, malonyl-CoA, and NADPH^16,17^. Whole-body knockout of FAS in mice causes embryonic lethality, confirming its essential role in early tissue development and embryogenesis^18^. However, in adult tissue, FAS was traditionally viewed as a housekeeping enzyme that contributed to hepatic triglyceride production for energy storage during nutrient-replete conditions and promoted β-oxidation of fatty acids through activation of PPARα under nutrient-deficient conditions^19^. But recent studies confirm a broader role for FAS in vascular tissue regulation. For example, FAS content is critical for endothelial cell nitric-oxide synthase (eNOS) signaling and cellular barrier function, and is decreased in the aortic tissue of *Db/Db* mice^20^. In macrophages, FAS is essential for prevention of diet-induced insulin resistance and recruitment of macrophages to adipose tissue^21^. Our study builds upon this body of knowledge and demonstrates that FAS content in peripheral arterial tissue is variable depending on disease severity, and also correlates with serum cFAS content. Interestingly, our study did not find any correlation between cFAS and FFAs in femoral plaque specimens, including SFA 16:0 and 18:0. Prior studies demonstrated that dietary intake and T2D can independently impact FFA content in vivo^22–24^, but whether this is directed by FAS-mediated de novo synthesis of SFAs in the peripheral arterial tissue remains to be elucidated.

In this study we observed elevated serum cFAS content in patients with CLTI, but no difference in serum cFAS enzyme activity. While it is unknown whether increased cFAS content or its biochemical enzymatic substrates in the serum are primarily responsible for conferring the presumed increased risk of peripheral arterial atheroprogression, our findings suggest serum cFAS of patients with advanced PAD is biochemically active. Similarly, other studies have implicated serum cFAS as a biomarker for disease progression including various forms of human cancer and metastatic disease^25–28^. Future studies may further explore therapeutic targeting of cFAS enzyme activity, particularly in populations with increased cFAS content, such as patients with CLTI as our data demonstrates.

Based on our prior findings that serum cFAS co-immunoprecipitated with ApoB in LDL cholesterol fraction from patients with carotid artery stenosis^13^, we hypothesized that FAS in arterial tissue may also co-localize with ApoB in patients with CLTI. Indeed, co-localization of FAS and ApoB staining appeared most notable in the arterial media where lipid cores emerge leading to atheroprogression^15^. These findings support our previously reported model that FAS produced by the liver is extruded into the blood stream in a semi-soluble form and conjugated to ApoB in non-HDL cholesterol particles such as VLDL and LDL^13^. Ultimately, intravascular LDL particles traverse the endothelium along with their cFAS cargo and deliver it to the sub-endothelial media layer to contribute to accelerated plaque formation. Additional studies are underway to further confirm this proposed molecular mechanism for peripheral atheroprogression.

We acknowledge that our study has limitations. First, this is a cross-sectional study which allowed us to evaluate serum cFAS as a marker for CLTI. Future studies using prospectively recruited study subjects, including health volunteers with no evidence of vascular disease, will be better suited to determine sensitivity and specificity of cFAS as an indicative biomarker that can predict disease progression. Second, due to the relatively small sample size, our study was not have sufficiently powered to evaluate differences in cFAS in patients with variable severity of PAD (different Rutherford classes and patients with no PAD) as well as other important comorbidities such as CKD. Further studies using a larger cohort may provide better accuracy and validity of our findings, and also provide additional information regarding how the extent of inflow versus outflow disease in patients with CLTI impacts serum cFAS levels. Finally, patient characteristics relied on chart review which may inadvertently contain misclassification errors. We attempted to set clearly defined criteria and revisited patient charts to confirm the quality and accuracy of the data to the best of our ability.

In conclusion, we found that serum cFAS content was significantly associated with CLTI, independent of concomitant diagnosis of T2D and smoking status. Using serum cFAS content, T2D, and smoking status provided high accuracy in distinguishing patients with CLTI. Furthermore, we demonstrated that serum cFAS content positively correlated with FAS content in femoral artery plaque tissue, and that FAS and ApoB co-localized in peripheral arterial wall medial layer. Our findings suggest that higher serum FAS may lead to altered FAS content in peripheral arterial tissue and impact atheroprogression in patients with CLTI. Future prospective studies may be able to determine whether serum cFAS content is a reliable serum-based diagnostic and predictive biomarker for patients with advanced PAD and CLTI.

## Methods

All methods were performed in accordance with institutional guidelines and regulations. All experimental protocols were approved by Institutional Review Board (IRB) at Washington University School of Medicine in St. Louis.

### Serum and tissue samples

We analyzed preoperative fasting serum from patients enrolled in an IRB-approved Vascular Surgery Biobank (see Supplementary information). Consecutive adults (aged ≥ 18 years) who underwent planned elective vascular surgery operative interventions from October 2014 to September 2016 were included. Individuals with CLTI who were undergoing lower extremity revascularization procedures were included in the analysis. Individuals without CLTI who were undergoing operative intervention for lower extremity revascularization, abdominal aortic aneurysm repair, carotid stenosis repair, neurogenic thoracic outlet syndrome (TOS) repair, or venous stripping or ablation were also included as non-CLTI controls. In a subset of patients with CLTI, intraoperative femoral artery endarterectomy plaque specimens and peripheral arterial segments from amputated lower extremities were also collected. All patients provided written informed consent to participate in this study.

### Variables of interest

We collected patient-level data such as demographics, disease severity, comorbidities, and medications from chart review. Severity of PAD was categorized as CLTI (Rutherford classes 4–6) and non-CLTI (Rutherford classes 0–3 and no PAD)^4^. Using CLTI as dependent variable, we evaluated serum cFAS, patient age, sex, race, comorbidities including T2D, insulin-dependency, and DCSI score^29^ (see Supplementary information). Due to the high co-occurrence of CLTI with T2D, we also analyzed a secondary model using T2D as a dependent variable.

### cFAS content and enzyme activity

Content of cFAS in the serum and FAS in lower extremity arterial plaque specimens were analyzed using commercially available human FAS ELISA Kit according to manufacturer’s recommendations (Aviva Systems Biology Corporation, San Diego, CA; see Supplementary information).

cFAS enzyme activity was evaluated using a modification of a previously described assay^30^. Serum concentrations were evaluated in batches using a Bradford Protein Assay (Bio-Rad Laboratories, Inc., Hercules, CA), and variable amounts of serum (5 to 30 μL) standardized by protein concentration were added to assay buffer (0.4 mL of 1 mol/L potassium phosphate buffer, 0.08 mL of 50 mmol/L EDTA, 0.08 mL of 50 mmol/L DTT, 0.6 mL of 1.1 mmol/L NADPH, and 0.24 mL of 1 mM acetyl CoA) and distilled water to a total volume of 90 μL. Finally, 10 μL of malonyl CoA was added and the rate of NADPH oxidation followed at 340 nm with a spectrophotometer. Mouse liver specimen was used positive control. FAS enzyme activity values were reported as picomole of NADPH consumed. Data quality control measures were used to evaluate consistency in results and accuracy.

### Free fatty acid in femoral plaque of patients with severe PAD

We measured saturated and unsaturated free fatty acid (FFA) levels in femoral plaque of patients with severe PAD (Rutherford classes 3–5) who are typically candidates for femoral endarterectomy procedures. Modified Bligh-Dyer method was performed as previously described (see Supplementary information)^13,31^. Briefly, plaque tissue was homogenized with lysis buffer prior to the addition of lipid internal standard solution. After phase separation, samples were extracted, vaporized to recover crystallized lipid, and analyzed with mass spectrometry. Each sample was evaluated at least in duplicate.

### Co-localization of FAS and ApoB in peripheral arterial segments

We collected lower extremity arterial segments from amputated lower extremities of patients with CLTI and T2D and withou T2D. Popliteal, anterior tibial, posterior tibial, and peroneal arterial segments were evaluated with immunofluorescent (IF) staining for FAS and ApoB (see Supplementary information). To quantify the proportion of FAS and ApoB signals within the arterial wall, images of stained arterial segments were digitally separated into intima (beginning of arterial wall to end of internal elastic lamina), media (between internal and external elastic lamina), and adventitia (external elastic lamina to end of arterial wall) using ImageJ software^32^. Pixel threshold for hue was set at red 0–20 for FAS, green 46–120 for ApoB, and yellow 21–45 for co-localization. Pixels beyond the hue threshold were defined as non-colocalized signals. Extraneous autofluorescence (beyond the arterial wall) were excluded from quantification. Signals were reported as a proportion of total signals.

### Statistical analysis

We summarized continuous variables with medians and interquartile ranges, and categorical variables with frequencies and percentages. Group comparisons were performed with Mann–Whitney U-test, Chi-square, or Fisher’s Exact test accordingly.

To evaluate FAS as a marker for CLTI, we performed multivariable logistic regression using CLTI as a dependent variable. Independent variables that were clinically important and statistically significant based on univariable analysis were included in logistic regression. No more than five independent variables were selected at a time using backward elimination. Sensitivity analysis was performed with ordinal logistic regression and excluded patients without PAD. To assess classification accuracy, we analyzed AUC^33^. AUC values closer to 1 indicate a greater accuracy in distinguishing outcome of interest. To evaluate co-localization of FAS and ApoB, co-immunofluorescent staining signals were analyzed using two-way analysis of variance (ANOVA). If *F* test was significant, Tukey’s test identified the arterial layer that differed from the others.

All tests were two-sided and *P* < 0.05 was considered significant. Hosmer–Lemeshow * χ*^2^ test with *P* > 0.05 indicates appropriate fit. All statistical analyses were performed with SAS Software version 9.4 (SAS Institute, Cary, NC). All graphs were generated with Stata 16.1 (StataCorp, College Station, TX).

## Supplementary Information

Supplementary Information.

## Data Availability

The datasets generated during the current study are not publicly available due to the protection of patient privacy but are available from the corresponding author on reasonable request.
