# Supplementary material for: Prevalence of elevated serum fatty acid synthase in chronic limb-threatening ischemia
Source: Sci Rep. 2021 Sep 29;11:19272. doi: 10.1038/s41598-021-98479-7 (PMC8481229; doi:10.1038/s41598-021-98479-7)
Supplement: Supplementary file 1 — Supplementary Information. [file 41598_2021_98479_MOESM1_ESM.docx]

**SUPPLEMENTARY METHODS**

**Vascular Surgery Biobank protocol**

Patients ≥18-years-old were eligible to be prospectively enrolled for collection of preoperative fasting serum and plaque tissue. Eligible procedures included carotid endarterectomy or stenting, upper or lower extremity peripheral artery bypass or percutaneous endovascular arterial repair with angioplasty and/or stenting, placement of inferior vena cava filter, thoracic outlet decompression, fistulograms, dialysis access procedure, venous stab phlebotomy, open or endovascular aortic aneurysm repair, and upper or lower extremity amputation. Patients under age 18 and those who were unable to provide written informed consent were excluded. Procedures excluded were subclavian bypass, aortic dissection repair, ruptured aortic aneurysm repair, embolectomy/thrombectomy, revascularization for acute limb ischemia, carotid body tumor, removal of infected bypass graft, removal and/or ligation of dialysis access, and any procedure performed for vascular trauma. All patients provided written informed consent prior to study participation. All study procedures were approved by the Washington University Institutional Review Board.

**Data collection**

Collection of patient characteristics included demographics (age, sex, and race), clinical (type of procedure, weight, height, and ankle brachial index), and comorbidities (type 2 diabetes mellitus (T2D), peripheral artery disease (PAD), chronic kidney disease (CKD), hypertension (HTN), hyperlipidemia, coronary artery disease (CAD), congestive heart failure (CHF), carotid artery stenosis (CAR), chronic obstructive pulmonary disease (COPD), insulin use, and smoking status). All patients with PAD were clinically diagnosed according to Rutherford chronic limb ischemia classification(4) based on clinical symptoms, physical exam, and non-invasive Doppler testing performed within 6 months prior to procedure. Chronic limb-threatening ischemia was classified as Rutherford classes 4-6 based on chart review (based on clinical symptoms, physical exam findings, and ankle-brachial index or toe-brachial index). Smoking status were categorized as never or former smokers (last smoked >12 months ago), and current smoker (last smoked within 12 months). CKD was defined as estimated glomerular filtration rate (eGFR) <60$mL/min/1.73m^{2}$ according to CKD-EPI formula, or eGFR ≥60 $mL/min/1.73m^{2}$ with comorbid diabetes complications severity index (DCSI) score of ≥5 to account for potential false elevation of eGFR in patients with T2D (16,34,35). CAD included stable and unstable angina or history of myocardial infarction. CAR included symptomatic and asymptomatic carotid artery stenosis. CHF included NYHA class I-IV. The types of procedure that patient underwent were categorized into the following: lower extremity (any lower extremity amputation, endovascular, or open surgical intervention), other peripheral vascular (carotid endarterectomy, carotid artery stenting, or dialysis-related procedure), aneurysm-related (abdominal aortic or thoracoabdominal aneurysms undergoing endovascular or open intervention), and non-arterial (neurogenic thoracic outlet syndrome and venous stab phlebectomy).

**cFAS Content and Enzyme Activity**

Content of cFAS in the serum and FAS in lower extremity arterial plaque specimens were analyzed using commercially available human FAS ELISA Kit according to manufacturer’s recommendations (Aviva Systems Biology Corporation, San Diego, CA). Samples were analyzed in batches and quality control measures were used to evaluate consistency and accuracy of results. Briefly, duplicate/triplicate serum or tissue samples were added to 96-well plate precoated with FAS-specific antibody, incubated for 2 hr at 37$℃$ and removed. A biotinylated detector antibody was added, incubated for 60 min at 37$℃$ and washed 3 times with wash buffer. Conjugate was added, incubated for 60 min at 37$℃$ and unbound conjugate was washed 5 times with wash buffer. Serum cFAS or tissue FAS levels were visualized by color change upon addition of color developing agent and acidic stop solution. Absorbance values were read at 450 nm with a spectrophotometer and cFAS or FAS concentrations were interpolated from the standard curve. Enzyme content values were reported as a ratio of total serum sample protein.

**Mass spectrometry analysis of free fatty acid in femoral plaque**

Modified Bligh-Dyer method was performed to extract free fatty acids (FFA) from 25µL of solubilized femoral plaque samples. d4-FFA (16:0) was used as internal standards for FFA. Internal standards were added to the samples before extraction.

The extracted FFA were further derivatized by amino methyl phenyl pyridium (AMPP) into FFA-AMPP derivatives to obtain high sensitivity in mass spectrometer. Four blank samples were derivatized by AMPP and were run with the study samples. The average signal of blank-AMPP for different FFA species was subtracted from the actual signal in the study sample to eliminate any interference introduced by reagents or glassware.

Measurement of lipids was performed with a Shimadzu 10A HPLC system and a Shimadzu SIL-20AC HT auto-sampler (GenTech Scientific, Arcade, NY) coupled to TSQ Quantum Ultra Triple Quadrupole Mass Spectrometer (Thermo Fisher Scientific, Waltham, MA) operated in SRM mode under ESI(+). Data processing was conducted with Xcalibur (Thermo Fisher Scientific, Waltham, MA).

Control samples were prepared by pooling the aliquots of the study samples and were used to monitor the instrument stability. Controls were injected six times in the beginning to stabilize the instrument and was injected between every six study samples. Only the lipid species with coefficient of variance < 15% in control samples were reported. The relative quantification of lipids was provided, and the data were reported as the peak area ratios of the analytes to the corresponding internal standards normalized to protein bovine serum albumin (BSA) standard. The relative quantification data generated in the same batch are appropriate to compare the change of an analyte in a test sample relative to other samples (e.g., control vs. treated, or samples in a time-course study).

**Immunofluorescent staining and imaging of FAS and ApoB in arterial segments**

Using a microtome, formalin-fixed paraffin-embedded blocks of minimally diseased lower extremity arterial segments from patients with CLTI were cut at 5 microns. After a water bath, tissue sections were transferred onto slides, dried overnight, and heated at 55$℃$ for 45 min to ensure tissue-slide attachment. Sections were deparaffinized with xylene twice for 5 min, rehydrated with 100% ethanol twice for 3 min, followed by 95% ethanol for 1 min, and rinsed with distilled water. For antigen retrieval, sodium citrate buffer was added to slides, heated to 95$℃$ for 10 min, and rinsed twice with phosphate-buffered saline (PBS) for 5 min. For blocking, 10% normal blocking serum from secondary antibody in 1-5% immunohistochemical-grade bovine serum albumin in PBS with Tween-20 (PBS-Tween 20) (Sigma-Aldrich, St. Louis, MO) were added, incubated for 15-60 min at room temperature, and removed. Primary antibodies for FAS (mouse monoclonal SC-48357; Santa Cruz Biotechnology, Dallas, TX) were added, incubated for 1 hr at room temperature, and rinsed 3 times with PBS-Tween 20 for 2 min. Secondary antibodies were added (donkey anti-mouse, 488 Green fluor; Fisher Scientific, Waltham, MA), incubated for 20-30 min at room temperature and rinsed 3 times with PBS-Tween 20 for 2 min. The above steps were repeated for primary antibodies for ApoB (rabbit polyclonal 20578-1-AP; Proteintech, Rosemont, IL), followed by secondary antibodies (donkey anti-rabbit, 555 Red fluor; Fisher Scientific, Waltham, MA). Counter stain with Flouroshield 4′,6-diamidino-2-phenylindole (DAPI) was added to the tissue to provide contrast against immunofluorescence (Sigma-Aldrich, St. Louis, MO). Slide images were captured using NanoZoomer 2.0-HT (Hamamatsu Corporation, Bridgewater, NJ).

**SUPPLEMENTARY TABLES**

**Supplementary Table 1.** Multivariable logistic regression with CLTI as dependent variable.

| Independent Variables | Odds Ratio (95% CI) |
| --- | --- |
| AUC 0.83 (95% CI, 0.74 – 0.92) | |
| cFAS content | 1.17 (1.04 – 1.31) |
| T2D | 5.22 (1.77 – 15.4) |
| Current smokers | 3.53 (1.19 – 10.5) |
| AUC 0.84 (95% CI, 0.76 – 0.93) | |
| cFAS content | 1.16 (1.02 – 1.31) |
| T2D | 3.70 (1.21 – 11.3) |
| Current smokers | 3.32 (1.11 – 9.96) |
| Age | 1.04 (0.99 – 1.09) |
| Male | 0.60 (0.18 – 2.0) |
| Female | 1.68 (0.50-5.61) |

AUC: Area under the receiver operating characteristics curve, cFAS: Serum circulating fatty acid synthase, T2D: Type 2 diabetes mellitus.

**Supplementary Table 2.** Ordinal logistic regression for patients with Rutherford classes 0-6 (n=59).

| Rutherford class | MLE (log odds) | P value |
| --- | --- | --- |
| Intercept (6 vs 0-5) | ‒3.19 | <.001 |
| Intercept (5-6 vs 0-4) | ‒1.91 | <.001 |
| Intercept (4-6 vs 0-3) | ‒0.42 | 0.27 |
| Intercept (3-6 vs 0-2) | 1.81 | <.001 |
| Intercept (2-6 vs 0) | 2.28 | <.001 |
| cFAS content | 0.10 | 0.016 |
| T2D | 1.25 | 0.013 |

FAS: Fatty acid synthase. T2D: Type 2 diabetes mellitus. MLE: Maximum likelihood estimate.

**Supplementary Table 3.** Baseline characteristics of patients with T2D who underwent serum analysis for FAS content.

| Characteristic | All Patients  n=87 | T2D  n=32 | No T2D  n=55 | P Value |
| --- | --- | --- | --- | --- |
| cFAS content | 4.11 (2.29-7.48) | 6.69 (2.86-9.28) | 3.32 (2.13-5.89) | .010* |
| Age, median – years | 62 (49-68) | 65.5 (59-69.5) | 58 (30-67) | <.001* |
| Sex – no. (%) |  |  |  | .78† |
| Male | 56 (64.4) | 20 (62.5) | 36 (65.5) |  |
| Female | 31 (35.6) | 12 (37.5) | 19 (34.5) |  |
| BMI, median – $\boldsymbol{kg}\boldsymbol{/}\boldsymbol{m}^{\boldsymbol{2}}$ | 29.4 (23.5-33.1) | 31.9 (27.2-36) | 27.4 (22.6-30.2) | .006* |
| Race – no. (%) |  |  |  | .018† |
| Caucasian | 71 (81.6) | 22 (68.8) | 49 (89.1) |  |
| African American | 16 (18.4) | 10 (31.2) | 6 (10.9) |  |
| PAD status – no. (%) |  |  |  | <.0001† |
| No PAD | 28 (32.2) | 2 (6.3) | 26 (47.3) |  |
| Rutherford 0-3 | 27 (31) | 10 (31.2) | 17 (30.9) |  |
| Rutherford 4-6 | 32 (36.8) | 20 (62.5) | 12 (21.8) |  |
| T2D – no. (%) | 32 (36.8) |  |  | N/A |
| DCSI score ≥5 | 22 (68.8) | 22 (68.8) | 0 |  |
| Insulin use | 15 (46.9) | 15 (46.9) | 0 |  |
| CKD – no. (%) | 33 (37.9) | 25 (78.1) | 8 (14.6) | <.0001† |
| CKD 1-3 | 28 (84.8) | 21 (84) | 7 (87.5) |  |
| CKD 4-5 | 5 (15.2) | 4 (16) | 1 (12.5) |  |
| Hyperlipidemia – no. (%) | 40 (46) | 20 (62.5) | 20 (36.4) | .018† |
| HTN – no. (%) | 55 (63.2) | 29 (90.6) | 26 (47.3) | <.0001† |
| CHF – no. (%) | 13 (14.9) | 8 (25) | 5 (9.1) | .062‡ |
| CAD – no. (%) | 26 (29.9) | 14 (43.8) | 12 (21.8) | .031† |
| CAR – no. (%) | 11 (12.6) | 5 (15.6) | 6 (10.9) | .52‡ |
| COPD – no. (%) | 14 (16.1) | 6 (18.8) | 8 (14.5) | .61† |
| Smoking status – no. (%) |  |  |  | .65† |
| Never/Former | 57 (65.5) | 20 (62.5) | 37 (67.3) |  |
| Current | 30 (34.5) | 12 (37.5) | 18 (32.7) |  |
| Procedure – no. (%) |  |  |  | N/A |
| TOS/venous stab | 20 (23) | 0 | 20 (36.4) |  |
| AAA/TAA repair | 17 (19.5) | 3 (9.4) | 14 (25.5) |  |
| CEA/CAS/dialysis access | 6 (6.9) | 4 (12.5) | 2 (3.6) |  |
| LE interventions | 44 (50.6) | 25 (78.1) | 19 (34.5) |  |

AAA: Abdominal aortic aneurysm. BMI: Body mass index $(kg/m^{2})$. CAR: Carotid artery stenosis. CAS: Carotid artery stenting. CEA: Carotid endarterectomy. cFAS: Serum circulating fatty acid synthase. CHF: Congestive heart failure. CKD: Chronic kidney disease. CLTI: Chronic limb-threatening ischemia. COPD: Chronic obstructive pulmonary disease. Current smoker: Last smoked within 12 months of vascular procedure. DCSI: Diabetes complications severity index. Former smoker: Last smoked >12 months before procedure. HTN: Hypertension. LE interventions: Lower extremity interventions included endovascular or open procedures and amputation. PAD: Peripheral artery disease. T2D: Type 2 diabetes mellitus. TAA: Thoracic aortic aneurysm. TOS: thoracic outlet syndrome. N/A: Not applicable. Continuous variables are presented as medians with interquartile ranges. Categorical variables are presented as frequencies and percentages. *Mann-Whitney U-test. † Chi-square test. ‡ Fisher’s Exact test.

**Supplementary Table 4.** Multivariable logistic regression with T2D as dependent variable.

| Independent Variables | Odds Ratio (95% CI) |
| --- | --- |
| AUC 0.896 (95% CI, 0.83 – 0.96) | |
| Age | 1.06 (1.01 – 1.11) |
| Male | 0.18 (0.04 – 0.83) |
| Female | 5.56 (1.20-25.8) |
| CKD | 24 (5.9 – 98.2) |
| AUC 0.899 (95% CI, 0.83 – 0.97) | |
| Age | 1.05 (1.00 – 1.11) |
| Male | 0.18 (0.04 – 0.86) |
| Female | 5.59 (1.16-27.0) |
| CKD | 25.4 (5.95 – 108) |
| cFAS content | 1.06 (0.95 – 1.19) |

AUC: Area under the receiver operating characteristics curve, cFAS: Serum circulating fatty acid synthase, T2D: Type 2 diabetes mellitus. CKD: Chronic kidney disease.

**Supplementary Table 5.** Baseline characteristics of patients who underwent analysis for serum and femoral endarterectomy plaque FAS content.

| Characteristics | CLTI  n=14 | No CLTI n=9 | P value | T2D  n=10 | No T2D  n=13 | P value |
| --- | --- | --- | --- | --- | --- | --- |
| Age, years | 64.5  (62-70) | 60  (59-62) | .018* | 62.5  (58-66) | 63  (61-68) | .28* |
| Sex |  |  | .18† |  |  | .02† |
| Male | 8 | 8 |  | 4 | 12 |  |
| Female | 6 | 1 |  | 6 | 1 |  |
| PAD |  |  | n/a |  |  | .53† |
| Rutherford 3 | n/a | 9 |  | 3 | 6 |  |
| Rutherford 4 | 13 | n/a |  | 6 | 7 |  |
| Rutherford 5 | 1 | n/a |  | 1 | 0 |  |
| T2D | 7 | 3 | .67† | 10 | n/a |  |
| DCSI ≥5 | 4 | 1 |  | 5 | n/a |  |
| DCSI <5 | 3 | 2 |  | 5 | n/a |  |
| cFAS content | 0.9  (0.77-1.36) | 1.09  (1.02-1.32) | .26* | 1.23  (0.94-1.98) | 1  (0.76-1.1) | .047* |
| Plaque FAS content | 9.91  (6.95-12.21) | 10.58  (8.98-11.83) | .75* | 13.23  (9.81-13.51) | 7.3  (5.01-11.41) | .013* |

CLTI: Chronic limb-threatening ischemia. T2D: Type 2 diabetes mellitus. DCSI: Diabetes complications severity index. N/A: Not applicable. Categorical variables are presented as frequencies. Continuous variables are presented as medians and interquartile ranges. *Mann-Whitney U-test. †Fisher’s Exact test.

**Supplementary Table 6.** Comparison of free fatty acid and FAS content in femoral endarterectomy plaque specimens from patients with severe PAD (n=22).

| Free Fatty Acid | Simple Linear  P value | Multiple Linear  P value |
| --- | --- | --- |
| SFA:UFA Ratio | .143 | .344 |
| Total SFA | .414 | .645 |
| Total UFA | .564 | .840 |
| SFA 16:0 (palmitic acid) | .364 | .589 |
| SFA 17:0 (margaric acid) | .064 | .184 |
| SFA 18:0 (stearic acid) | .584 | .790 |
| SFA 20:0 (arachidic acid) | .118 | .295 |
| UFA 18:1 (vaccenic acid) | .126 | .278 |
| UFA 18:2 (linoleic acid) | .839 | .959 |
| UFA 20:4 (arachidonic acid) | .676 | .913 |
| UFA 22:1 (erucic acid) | .118 | .295 |

SFA: Saturated fatty acid. UFA: Unsaturated fatty acid. Simple linear regression compared FFA with FAS content. Multiple linear regression compared FFA with FAS content and T2D.

**Supplementary Table 7.** Comparison of free fatty acid content in femoral endarterectomy plaque and serum FAS content in patients with severe PAD (n=16).

| Free Fatty Acid | Simple Linear  P value | Multiple Linear  P value |
| --- | --- | --- |
| SFA:UFA Ratio | .924 | .865 |
| Total SFA | .671 | .900 |
| Total UFA | .431 | .742 |
| SFA 16:0 (palmitic acid) | .783 | .902 |
| SFA 17:0 (margaric acid) | .907 | .801 |
| SFA 18:0 (stearic acid) | .521 | .815 |
| SFA 20:0 (arachidic acid) | .941 | .710 |
| UFA 18:1 (vaccenic acid) | .958 | .986 |
| UFA 18:2 (linoleic acid) | .308 | .595 |
| UFA 20:4 (arachidonic acid) | .433 | .744 |
| UFA 22:1 (erucic acid) | .941 | .710 |

SFA: Saturated fatty acid. UFA: Unsaturated fatty acid. Simple linear regression compared FFA with serum FAS content. Multiple linear regression compared FFA with serum FAS content and T2D.

**SUPPLEMENTARY FIGURES**

**
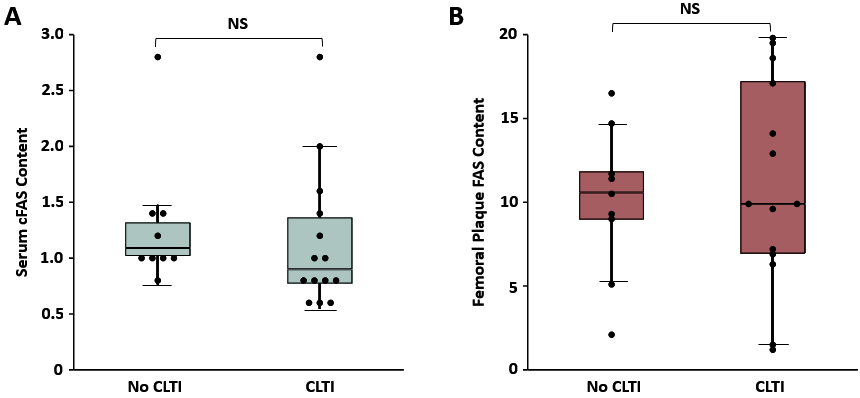
**

**Supplementary Figure 1. FAS content in serum and femoral plaques of patients with severe PAD**

Among 14 patients with CLTI (Rutherford 4 and 5) and 9 without CLTI (Rutherford 3), there was no significant difference in (**A**) serum FAS content (0.9, IQR 0.77-1.36 *vs.* 1.09, IQR 1.02-1.32; *P*=.26) or (**B**) plaque FAS content (9.91, IQR 6.95-12.21 *vs.* 10.58, IQR 8.98-11.83; *P* =.75). Based on Mann-Whitney U-test. Boxplot indicates interquartile range (IQR), median (horizontal line within box), and 1.5 IQR of upper quartile (top whisker) and lower quartile (bottom whisker). FAS content values were reported as ratios of total serum or plaque protein. NS: Not significant.

**
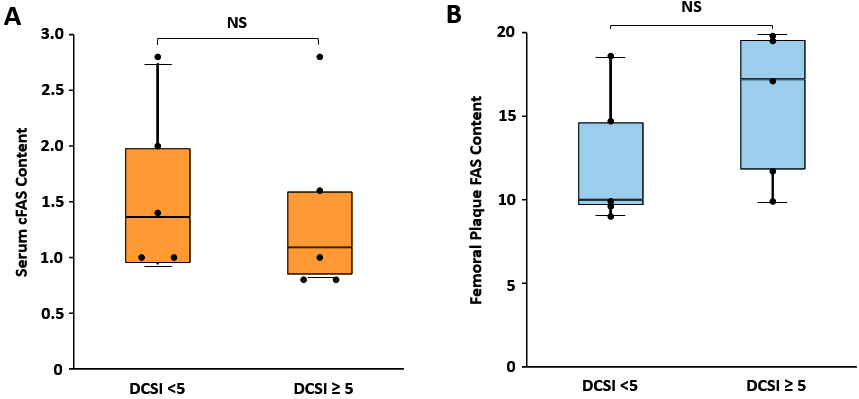
**

**Supplementary Figure 2. FAS content in serum and femoral plaque of T2D patients with DCSI scores**

In patients with T2D, there was no difference in (**A**) serum (1.60±0.77 *vs*. 1.43±0.81, *P*=.6) or (**B**) plaque FAS content (12.4±4.1 *vs*. 15.7±4.6, *P*=.17) between patients with DCSI score <5 (n=5) and DCSI score ≥5 (n=5). Boxplot indicates interquartile range (IQR), median (horizontal line within box), and 1.5 IQR of upper quartile (top whisker) and lower quartile (bottom whisker). FAS content values were reported as ratios of total serum or plaque protein. NS: Not significant. DCSI: Diabetes Clinical Severity Index
